# Stress Relaxation Behavior of Additively Manufactured Polylactic Acid (PLA)

**DOI:** 10.3390/ma15103509

**Published:** 2022-05-13

**Authors:** Alcide Bertocco, Matteo Bruno, Enrico Armentani, Luca Esposito, Michele Perrella

**Affiliations:** Department of Chemical, Materials and Production Engineering, University of Naples Federico II, Piazzale V. Tecchio 80, 80125 Naples, Italy; matteo.bruno@unina.it (M.B.); enrico.armentani@unina.it (E.A.); luca.esposito2@unina.it (L.E.); michele.perrella@unina.it (M.P.)

**Keywords:** additive manufacturing, stress relaxation, viscoelasticity, analytical fitting, experimental mechanics, polylactic acid (PLA)

## Abstract

In this work, the stress relaxation behavior of 3D printed PLA was experimentally investigated and analytically modeled. First, a quasi-static tensile characterization of additively manufactured samples was conducted by considering the effect of printing parameters like the material infill orientation and the outer wall presence. The effect of two thermal conditioning treatments on the material tensile properties was also investigated. Successively, stress relaxation tests were conducted, on both treated and unconditioned specimens, undergoing three different strains levels. Analytical predictive models of the viscous behavior of additive manufactured material were compared, highlighting and discussing the effects of considered printing parameters.

## 1. Introduction

Additive manufacturing (AM), or 3D printing, is an advanced technology that enables high-accuracy and low-cost production of physical models and structures of complex geometry. In the last few years, AM significantly evolved in a growing market. Additive-manufactured parts are built layer by layer with deposition of material according to 3D digital design [1,2,3,4]. AM technology is currently adopted in a wide range of engineering applications such as mechanical, biomedical, construction, aerospace, and food industries as well as in academic research [5,6,7,8,9]. Currently, many different technologies are available in the AM field and can be classified by means of the energy source or the way the material is joined, for instance, by using a binder, laser or heated nozzle. A classification is also possible by group of materials processed, such as plastics, metals or ceramics [10]. The most common materials used in AM are certainly plastics and polymers in general; nevertheless, the strong technological development has allowed also metallic and ceramic materials to become of interest in this field. Table 1 shows the different materials that can be used in relation to the various technological processes described. Among polymers, polylactic acid (PLA), also known as polylactide, is attracting increasing interest from industry and researchers. PLA is a biodegradable thermoplastic polyester derived from totally renewable resources such as sugar beets and corn [11]. In specific conditions [12], the decomposition of PLA into water, carbon dioxide and humus (the black organic material in soil) can be obtained. Furthermore, PLA shows interesting mechanical properties such as high stiffness and high strength if compared to many synthetic polymers [13]. Physical and mechanical properties of PLA are extensively discussed in [14]. Therefore, PLA is proving to be a potential alternative to replace petroleum-derived polymers [12]. It is used in a variety of bio-medical applications as well such as dialysis media, porous scaffolds, bone-fixation devices, interference screws, drug-eluting stents, sutures, and suture anchors [15,16,17]. It is noteworthy that untreated PLA has mechanical properties heavily dependent on temperature and, for this reason, its application is preferable in low-temperature solutions, usually up to 60 °C [18]. On the contrary, an annealing thermal treatment (between glass transition and melt temperatures) could enhance the mechanical properties of FDM–PLA parts [19].

Fused deposition modeling (FDM) is the most popular and affordable AM extrusion-based method to manufacture polymer-based structures. Because of its extensive use, a thorough understanding of the influence of the manufacturing process on the mechanical properties of PLA material is crucial. The quality of products fabricated by the FDM process is usually affected from surface roughness, poor precision and low strength [20]. An opportune selection of printing parameters, such as temperature, printing speed, wall thickness and layer height, can significantly improve the overall printed samples quality [21,22]. The mechanical behavior of polymeric structures is mainly governed by time dependent rheological effects, and therefore, the prediction of the inelastic mechanical behavior, in terms of monotonic and cycling loading, as well as creep and relaxation, is of great importance [23,24]. The viscoelastic behavior of polymers strongly influences the optimal choice of their fields of application. The viscoelasticity of materials is exhibited in different ways, such as the progressive deformation of a material sample under constant stress, i.e., creep behavior, and the gradual reduction in force when the sample is subject to a constant strain, i.e., stress relaxation. In general, viscoelasticity is a phenomenon associated with time-dependent material response [25]. Biodegradable polymers can undergo failure much before the anticipated yielding and ultimate tensile strength, due to its viscous nature, which leads to the creep or relaxation rupture. For example, in Grabow et al. [26], significant creep deformation in PLA stents was reported when subjected to a constant load. Up to the proportionality limit, deformation occurring in the polymer specimen is similar to the uncoiling of a molecular chain. No intermolecular slippage is noticed, and strains are recoverable in this area but only after a certain period. The deformation that occurs beyond this proportionality limit is unrecoverable. These distortions occur due to the actual displacement of the molecules over each other, which causes permanent deformation [27,28]. In [29], the creep behavior of biodegradable PLA was analyzed, considering the effects of layer thickness and printing angle. Creep response was also modeled by using Burger model for predictive purposes. The static properties of printed PLA and their dependency on printing parameters have been extensively studied, and many works are available on these topics. On the other hand, although several papers have dealt with the investigation of creep behavior of printed PLA materials, the literature on stress relaxation response of PLA subject to thermal ageing is limited. In this work, an investigation of the viscoelastic behavior of the PLA as a function of the infill strategy is presented. First, the static characterization of PLA samples, printed using different infill orientations, was carried out. Afterwards, an extensive campaign of stress relaxation tests was conducted. Here, the influence of infill strategy was taken into account. Finally, analytical predictive models to describe the relaxation behavior of the PLA printed by FDM technology were described and compared. The presented results can be useful for expanding the use of PLA material in the 3D printing of products for further engineering sectors.

## 2. Materials and Methods

### 2.1. Experimental Methods

The experimental campaign was performed on samples printed with a PLA plus filament, by Zhuhai SUNLU Industrial Co., Zhuhai, China, whose nominal properties are reported in Table 2.

**Figure 1:**
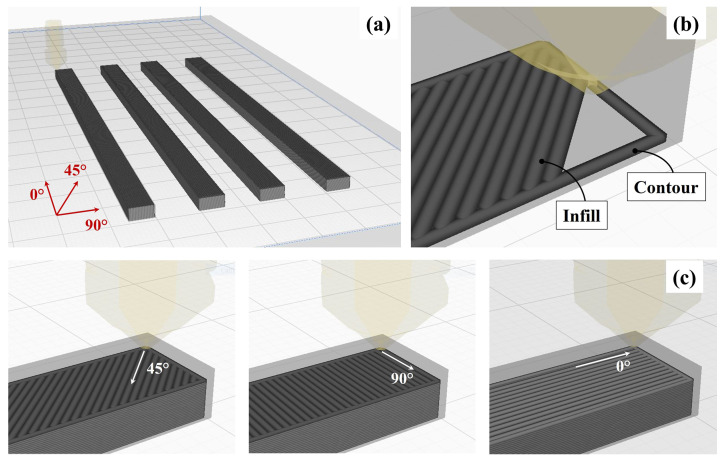
Schematic representation of specimens geometry (**a**), contour and infill lines (**b**) and considered printing direction (**c**).

A commercial FDM linear Cartesian machine, Artilery Sidewinter X1 (Shenzhen Yuntuchuangzhi Technology Co., Ltd., Shenzhen, China), equipped with a direct extrusion system, was adopted to 3D print the material. It was extruded through a 0.4 mm-diameter nozzle with an extrusion temperature of 210 °C and a deposition velocity of 60 mm/s. Other critical printing parameters such as the bed plate temperature and the layers height were set to 50 °C and 0.1 mm, respectively. The manufacturing process was conducted in a controlled environment. Currently, there is no specific standard reference for additively manufactured polymers testing. Therefore, the experimental campaigns presented in this work were conducted by adopting a custom rectangular cross section sample, Figure 1a, with a length in the traction direction equals to 120 mm, a width of 6 mm and a thickness of 3 mm. The gauge length was set to 25 mm. The specimens were clamped using self-tightening wedge grips mounting specific jaws for polymers, thus avoiding specimen distortions due to overly hard clamping. Tests on specimens realized according to ASTM D638 standard were also performed, but the dog bone geometry seemed not suitable for the FDM samples printed with the selected parameters, due to the great deal of breakages in fillets close to the clamping area. A similar premature failure of ASTM D638 printed specimens was also highlighted in [30].

In a standard FDM process, the thermoplastic filament is melted in a liquefier and extruded through a nozzle onto the build platform according to the predetermined tool paths. Once a layer is completed, the extrusion apparatus is raised (or the build platform is lowered), and then the next layer is deposited on the previous one. The whole process is repeated until the entire part geometry is realized [31]. Generally, the FDM technology is characterized by the presence of contour lines, Figure 1b, enclosing the slice section, representing a key factor respect to the workpiece dimensional quality and geometrical accuracy [32]. Sets of specimens were printed with and without the outer wall, consisting of a single contour line, to investigate the effect of the contour on the mechanical behavior of additive manufactured material. In addition, to evaluate the influence of the printing orientation on the PLA mechanical response, three different printing infill directions were tested as schematically reported in Figure 1c. The considered infill orientations were $0^{\circ },45^{\circ }$ and $90^{\circ }$ respect to the traction direction.Every layer was printed using the same infill direction and automatic stacking strategy was not adopted. Moreover, in order to analyze the mechanical behavior of printed PLA under operative conditions, heat and cold thermal treatments were applied to specimens printed with the 0° infill direction, i.e., the configuration providing higher strength. The heating conditioning sequence was obtained by holding the samples for 8 h in an air-circulating oven at a temperature ($T_{h}$) of about the 80% of the material transition temperature $T_{g}$. The cooling treatment consisted of maintaining the specimens for 8 h in a laboratory freezer at constant $T_{c}=-15\;{}^{\circ}C$ temperature. The temperature levels selected for the thermal treatments were chosen as representative of operating conditions to simulate the natural aging of the material. In both cases, the specimens returned to room temperature in calm conditions.

Quasi-static tensile characterization was conducted to estimate the material elastic properties depending on the infill orientation, the contour influence and thermal conditioning. A servo-hydraulic testing machine Instron 8500 plus (Instron, Buckinghamshire, UK), with a 1 kN load cell, was adopted to execute tensile tests under displacement control. The average elastic modulus and the deformation correspondent to the maximum reached stress were estimated. Successively, experimental stress relaxation characterization of the additively manufactured PLA was executed under strain control. Constant tensile deformation $\epsilon_{o}$ was applied to the specimens and the corresponding engineering stress trend σ(t) was evaluated over an assigned dwell time Δt. In the present work, three straining values, respectively, equal to the 27%, 36% and 45% of the deformation at the maximum stress, were considered for the contoured 0°, 45° and 90° infill printed direction samples. The relaxation phenomenon was measured over a time Δt=500 s.

### 2.2. Analytical Methods

Three different predictive laws were adopted to model the viscoelastic behavior of PLA samples. A comparison of analytical modeling and an evaluation of the infill direction influence on the relaxation behavior was performed. All the experimental data from the three test strain levels for each infill printing direction were collected in a unique set to identify model parameters. The best data fitting was reported by means of the correlation coefficient. First, a rheological Maxwell model was implemented [33]. As reported in (1), the trend of the stress over the time depends on the imposed straining $\epsilon_{0}$ level and on the two parameters $A_{1}$ and $A_{2}$ which, respectively, represent the spring stiffness and the dashpot damping value.

(1) $$\sigma \left(t\right)=\epsilon_{0}\cdot A_{1}\cdot \epsilon^{-t/A_{2}}$$

Second, a more complex standard Linear Solid model [34] was implemented. In (2), the correspondent formulation for the stress evolution over the time, is reported.

(2) $$\sigma \left(t\right)=\epsilon_{0}\cdot \left[B_{1}+B_{2}\cdot \epsilon^{-t/B_{3}}\right]$$

**Figure 2:**
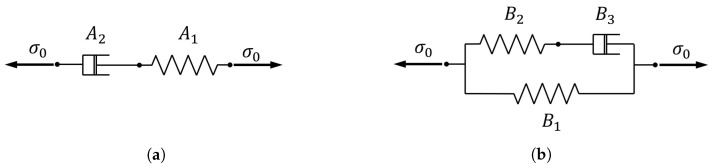
Scheme of the considered linear rheological models: (**a**) Maxwell model and (**b**) Linear Solid model.

Similar to the Maxwell approach, in the standard linear solid model, the stress is linearly dependent on the imposed strain level $\epsilon_{0}$ through three elements: a spring with stiffness $B_{2}$ and a dashpot with damping coefficient $B_{3}$ combined in parallel to a further spring of stiffness $B_{1}$. The representations of the above-mentioned Maxwell and Linear Solid rheological models are reported in Figure 2a,b, respectively.

Finally, the nonlinear *Findley* model was enforced [35]. According to (3), the evolution of stress over time results to be function of the initial stress value $\sigma_{0}$ at the time t=0, the stress-dependent fitting parameter $\sigma_{1}$, the fitting exponent *n* and of the material constant $t_{c}$ that was considered as unitary in the present study, according to literature [36,37].

(3) $$\sigma \left(t\right)=\sigma_{0}+\sigma_{1}\cdot {\left(t/t_{c}\right)}^{n}$$

Since the purpose of this work is the estimation of relaxation influence on the elastic properties, the presented analytical models were analyzed in terms of creep modulus E(t), obtained by dividing the stress over time σ(t) by the constant deformation value $\epsilon_{0}$, as reported in (4).

(4) $$E\left(t\right)=\frac{\sigma (t)}{\epsilon_{0}}$$

In the following Table 3, the resultant creep modulus expression and the fitting constants are reported for each considered model. It is worth noting that only the parameters $\sigma_{0}$ and $\sigma_{1}$ of Findley law modified their nomenclature in $E_{0}$ and *C* due to the division by remote strain.

Experimental curve fitting was achieved, implementing the creep modulus expression on the MatLab Curve Fitting Toolbox, adopting the non-linear least squares method with the trust region fit option enabled. The R-squared ($R^{2}$) values were compared to determine the most accurate model for the experimental data fitting. The identified law was adopted to appreciate the influence of the material infill direction, as well as the thermal conditioning, on the relaxation behavior of the tested 3D printed PLA.

## 3. Results and Discussion

### 3.1. Experimental Quasi-Static and Long-Term Behavior

With the purpose to estimate the elastic behavior of the AM material, three tensile tests were performed on each specimen type. The elastic modulus *E*, the tensile strength $\sigma^{*}$ and the correspondent deformation $\epsilon^{*}$ of the contoured specimen type were reported in Table 4 collected by the material infill direction.

**Figure 3:**
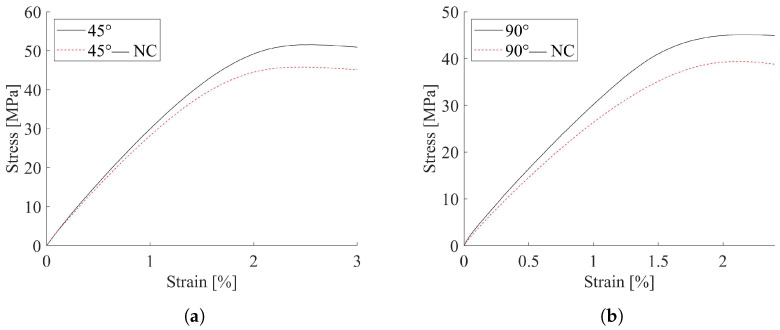
Contoured vs. not contoured specimens tensile curves; (**a**) 45° infill orientation; (**b**) 90° infill orientation.

The 0° specimens resulted in the higher values of the elastic modulus, strength and correspondent deformation at peak stress. The 45° samples presented a drop in the elastic modulus of about 5% and a reduction in strength of 10%, while the 90° specimens carried out a tensile strength lower than 21% with respect to the 0° direction. The effect of contour was estimated and representative results for the 45° and for 90° infill direction are, respectively, reported in Figure 3a,b. A comparison with the correspondent contoured specimen outcomes is also shown in the same figure. The different behavior could be related to the way the infill lines are loaded. Indeed, in the 0° specimens all the filament lines were aligned to the loading direction. Therefore, they were loaded along their axis. Instead, the 90° specimens were orthogonal to the load, thus the resultant interface between adjacent filaments was much more stressed than in the 0° specimens. Moreover, samples with an outer wall presented contour lines always oriented at 0°, i.e., the stiffer direction. Despite only one outer wall line being used, the experimental stress–strain curves of contoured 45° and 90° samples were considerably influenced by its presence. The hygroscopic nature of the PLA material [38] and manufacturing flaws can influence the behavior of printed samples. Within this context, thermal treatments, in particular cooling conditioning, could provide performance degradation.

A reduction in strength was noticeable and not influenced by the infill direction, with a drop of about 16% for both the considered directions, while negligible variations were appreciated in the elastic modulus. Furthermore, the influence of thermal treatments on the material elastic properties were estimated through tensile tests conducted on 0° conditioned specimens; the results are reported in Table 5.

**Figure 4:**
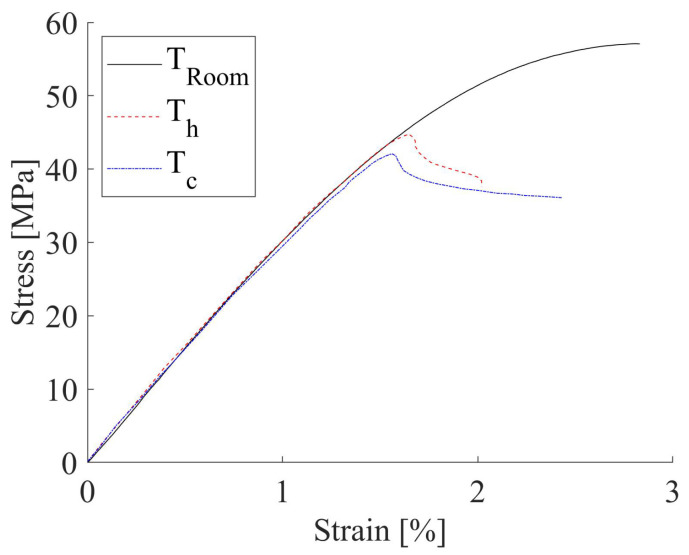
Tensile curves of 0° specimens thermally treated and unconditioned.

The quasi-static tensile behavior of thermal conditioned sets is graphically reported in Figure 4. In comparison with the untreated material, a fairly negligible difference (<3%) was detectable in elastic modulus, whilst a decay of about 25–29% in strength was noticed. The cooled specimens were demonstrated to have worse mechanical behavior.

**Figure 5:**
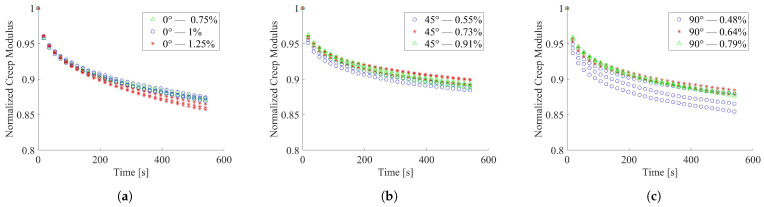
Normalized creep modulus over time at room temperature; (**a**) 0° infill orientation; (**b**) 45° infill orientation; (**c**) 90° infill orientation.

The stress relaxation behavior of printed PLA material was investigated and the results discussed in term of normalized creep modulus over time. The normalized creep modulus was computed as the ratio between the creep modulus (4) and the average elastic modulus above was presented. The relaxation results, for the imposed strain values, were reported collected by the infill printing orientation, in Figure 5a for the 0°, in Figure 5b for 45° and in Figure 5c for 90° infill orientation.

It is worth noting that samples printed with 90° orientation seem to exhibit non-linear viscoelastic behavior. This response could be ascribed to the way the material is loaded with respect to the infill lines orientation. The 90° samples lay orthogonally to the loading direction, and the sample’s response was much more influenced by the interface behavior of adjacent infill lines and eventual flaws.

**Figure 6:**
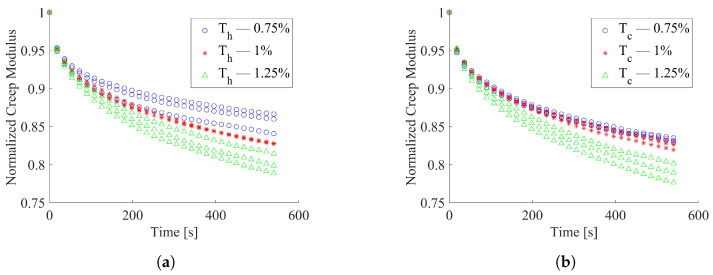
Normalized creep modulus over time for thermally conditioned 0° specimens; (**a**) Thermal conditioning T = 50 °C; (**b**) Thermal conditioning T = −15 °C.

The 0° and 45° tested specimens resulted in less dispersed data compared to that showed by the 90° specimens. The average creep modulus decay, in the investigated Δt, was estimated to be equal to about 13%, 11% and 13%, respectively, for 0° and 45° and 90°. The outcomes of relaxation tests on the thermally conditioned 0° specimens, here reported in Figure 6a with regard to heating treatment and in Figure 6b for those subject to cooling, showed higher data scatter than unconditioned ones. Both thermal treatments produced an increment of the creep modulus decay with respect to the unconditioned test outcomes.

### 3.2. Analytical Modeling of Stress Relaxation Response

The above-proposed analytical models were implemented to fit experimental data sets with all the adopted strain levels. In such a way, the effect of the infill direction on the material relaxation behavior was highlighted. To estimate the models’ fitting quality, the coefficient of determination (R-squared) values were computed and reported in Table 6. With respect to the Maxwell and Linear Solid relationships, the Findley model resulted in the higher average value of $R^{2}$, always over 0.85.

**Figure 7:**
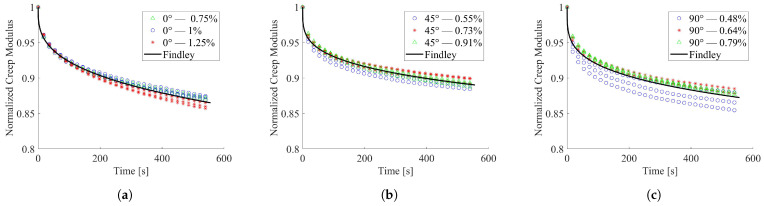
Trend of normalized creep modulus at room temperature; (**a**) 0° infill orientation; (**b**) 45° infill orientation; (**c**) 90° infill orientation.

A graphical representation of the Findley empirical model outputs and the experimental outcomes, in terms of normalized creep modulus, is presented in Figure 7a for 0°, in Figure 7b for 45°, and in Figure 7c for the 90° infill direction.

The same analytical fitting procedure was applied to the experimental relaxation results of thermally conditioned specimens. The $R^{2}$ values, carried out by the analyses of analytical models, are listed in Table 7. Furthermore, for the treated specimens, the Findley law showed a satisfactory prediction of response, with $R^{2}$ values always over 0.93.

Among the investigated predictive models, the Findley equation resulted in the best experimental data fitting, independent of the infill orientation and thermal treatment. Thus, it was selected for comparing the stress relaxation behavior of the untreated and thermally conditioned specimens with different infill orientations. The computed values of Findley model parameters are presented in Table 8 for all the considered testing conditions and manufacturing processes.

**Figure 8:**
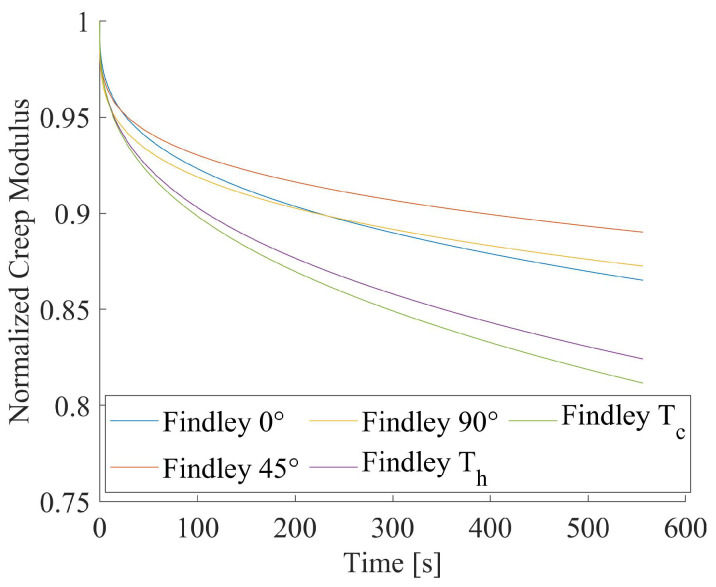
Comparison of stress relaxation curves by Findley model.

The comparison among the analytical curves for all the investigated cases is reported in Figure 8. It shows that the material relaxation behavior is hardly influenced by the infill printing direction. Indeed, the normalized creep modulus decreased in the range of 11–14%. From a practical point of view, a difference of about 3% can be considered negligible. The thermal conditioning procedures affected relaxation response of printed PLA, showing a stress decay greater than 45% with respect to the untreated samples.

## 4. Conclusions

In this study, three different printing infill directions (0°, 45° and 90°) were considered for evaluating the tensile behavior of printed PLA by FDM technique. The presence of a single-line outer wall was also analyzed. The tensile strength of contoured 0° and 45° specimens was higher than about 17% of the ones without an outer wall. From an operational point of view, the presence of the contour increases the dimensional accuracy of the product.

Secondly, the stress relaxation response was experimentally investigated under different uniaxial strain levels at room temperature. Stress decay ranging from 11% to 14% was acquired. Three analytical models were used for describing stress relaxation response: Maxwell equation, standard Linear Solid model and Findley law. Among them, the Findley empirical expression was confirmed as being the most suitable predicting the tested PLA.

Furthermore, two thermal conditioning procedures were considered for 0° samples in order to reproduce the natural ageing of materials. Both cooled and heated specimens provided a degradation of quasi-static and long-term material properties.

The presented outcomes pointed out the significance of stress relaxation effects in AM PLA structures. Thus, a preliminary predictive analysis should be considered to guarantee reliability over time of 3D printed parts in applications with imposed displacements, e.g., medical brace prostheses or screw joints.

## Figures and Tables

**Table 1:** Materials for additive manufacturing and related technological processes.

Technology	Polymers	Metals	Ceramics	Composites
Stereolithography	•			•
Digital light processing	•			
Multi-jet modeling	•			•
Fused deposition modeling	•			
Electron beam melting		•		
Selective laser sintering	•	•	•	•
Selective heat sintering	•			
Direct metal laser sintering		•		
Plaster-based 3D printing			•	•
Laminated object manufacture	•	•	•	•
Ultrasonic consolidation		•		
Laser metal deposition		•		•

**Table 2:** Nominal PLA filament properties provided by the supplier.

Filament Diameter [mm]	Tensile Strength [N]	Melt Flow Rate [g/10 min]	Transition Temperature T_g_ [°C]
1.75±0.02	108÷147	9÷11	62.5

**Table 3:** Considered analytical models creep modulus expression and fitting constants.

	Maxwell	Linear Solid	Findley
Creep Modulus	$A_{1}\cdot e^{-t/A_{2}}$	$B_{1}+B_{2}\cdot e^{-t/B_{3}}$	$E_{0}+C\cdot t^{n}$
Material Constants	$A_{1};A_{2}$	$B_{1};B_{2};B_{3}$	C;n

**Table 4:** Elastic characterization of contoured specimens.

Infll Direction	0°	45°	90°
*E* [MPa]	3045 ± 3	2914 ± 3	2932 ± 3
$\sigma^{*}$ [MPa]	60 ± 3	54 ± 3	47 ± 3
$\epsilon^{*}$	2.75%	2.05%	1.75%

**Table 5:** Elastic characterization of thermally conditioned 0° specimens.

Conditioning	*T_h_* = 50 °C	*T_c_* = −15 °C
*E* [MPa]	3005 ± 3	2990 ± 3
$\sigma^{*}$ [MPa]	46 ± 3	43 ± 3
$\epsilon^{*}$	1.56%	1.65%

**Table 6:** Analytical models R-squared values for the 0°, 45° and 90° infill orientation.

$R^{2}$	0°	45°	90°
Maxwell	0.87	0.80	0.73
Linear Solid	0.96	0.92	0.84
Findley	0.97	0.94	0.85

**Table 7:** R-squared values from data fitting of the thermally conditioned specimens.

$R^{2}$	$T_{h}$	$T_{c}$
Maxwell	0.87	0.80
Linear Solid	0.96	0.92
Findley	0.97	0.94

**Table 8:** Findley model parameters for the treated and unconditioned specimens.

	Specimen Type	C	*n*
Untreated	0°	−51.4605	0.3283
	45°	−60.0284	0.2650
	90°	−70.6612	0.2636
Treated	$T_{h}$	−59.1985	0.3462
	$T_{c}$	−57.7070	0.3604

## Data Availability

The data that support the findings of this study are available from the corresponding author upon reasonable request.

## Abbreviations

The following abbreviations are used in this manuscript:

PLA	Polylactic Acid
AM	Additive Manufacturing
FDM	Fused Deposition Modeling
